# Evolution of Seventh Cholera Pandemic and Origin of 1991 Epidemic, Latin America

**DOI:** 10.3201/eid1607.100131

**Published:** 2010-07

**Authors:** Connie Lam, Sophie Octavia, Peter Reeves, Lei Wang, Ruiting Lan

**Affiliations:** Author affiliations: University of New South Wales, Sydney, New South Wales, Australia (C. Lam, S. Octavia, R. Lan);; niversity of Sydney, Sydney (P. Reeves);; Nankai University, Tianjin, People’s Republic of China (L. Wang)

**Keywords:** Bacteria, Vibrio cholerae, enteric infections, cholera, single nucleotide polymorphisms, pandemic, Latin America, dispatch

## Abstract

Thirty single-nucleotide polymorphisms were used to track the spread of the seventh pandemic caused by *Vibrio cholerae*. Isolates from the 1991 epidemic in Latin America shared a profile with 1970s isolates from Africa, suggesting a possible origin in Africa. Data also showed that the observed genotypes spread easily and widely.

The seventh cholera pandemic began in 1961, and by 1966, it had affected most of Asia. Cholera incidence then decreased slightly until 1971, when an upsurge was observed in Africa and Europe, which had been free of cholera for >100 years (*1*). Cholera rates remained relatively low during the 1980s, with the disease confined to Asia and Africa. However, 2 major cholera outbreaks appeared in the 1990s: first, a resurgence of cholera in Africa, and, second, outbreaks that started in Peru became the first cholera epidemic in Latin America since 1895 (*2*). In addition, a novel serotype caused major outbreaks on the Indian subcontinent in 1992. That strain was referred to as O139 Bengal and was later shown to be a variant of the seventh pandemic clone with its replacement of the O antigen (*1*). Pulsed-field gel electrophoresis (*3*), amplified fragment length polymorphism analysis (*4*), and ribotyping (*1*) have been applied to seventh pandemic isolates but did not fully resolve the relationships of the various outbreaks. In this study, we used genome-wide single-nucleotide polymorphisms (SNPs) to track the evolution and spread of the seventh cholera pandemic, including the O139 Bengal strain.

## The Study

The availability of complete genome sequences of a pre–seventh pandemic isolate, M66–2 (*5*), a seventh pandemic isolate, N16961 (*6*), and the partial genome sequence of an O139 Bengal isolate, MO10 (*7*), enabled identification and use of SNPs as evolutionary markers in *Vibrio cholerae*. A set of 18 SNPs was chosen from 125 N16961 SNPs (*5*) and 12 SNPs selected from 59 identified by comparison of the N16961 and MO10 genome sequences. The SNPs selected were mostly from genes with known function and were distributed throughout the 2 chromosomes for the N16961 SNPs and the large chromosome for the MO10 SNPs. We have previously shown that recombinant regions could be identified by the differences in distribution of SNPs in such regions (*5*); for this study, only mutational SNPs were selected.

The 30 SNPs (Technical Appendix) were used to type a collection of 64 seventh pandemic *V. cholerae* isolates. SNPs were detected by using hairpin primer real-time PCR. SNP data for 3 complete *V. cholerae* genomes (M66–2, N16961, MJ-1236) and 4 partially sequenced genomes (MO10, RC9, B33, CIRS 101) (*7*) were obtained from the National Center for Biotechnology Information (Rockville, MD, USA) and included in the analysis. The 71 isolates were divided into 10 SNP profiles by using the 30 SNPs (Table A1). Three profiles were represented by 1 isolate only, whereas the remaining profiles contained 4–17 isolates. The Simpson index of diversity for all SNPs combined was 0.929.

A maximum-parsimony tree (Figure) was constructed to show the relationships of the SNP profiles. The tree was fully resolved with no reverse or parallel changes in the seventh pandemic isolates. The pre–seventh pandemic strains were used as an outgroup and placed at the base of the tree. Six groups could be distinguished, with each group containing SNP profiles differing by no more than 1 SNP. The ladderized tree shows the stepwise evolution of the SNP profiles and groups. Group I at the bottom of the tree originated in Indonesia in 1961. It contains mostly isolates from Asia from the 1960s but continued to be isolated in Southeast Asia. The other groups evolved sequentially. Group II contains isolates from Africa from the 1970s to the 1990s and all 4 isolates from Latin America; group III contains earlier 1970s isolates from Asia and 1980s isolates from Africa; group IV contains late 1970s and 1980s isolates from Asia only; while group V contains 1990s isolates from Asia and Africa. Group VI contains only O139 isolates with the same SNP profile.

**Figure:**
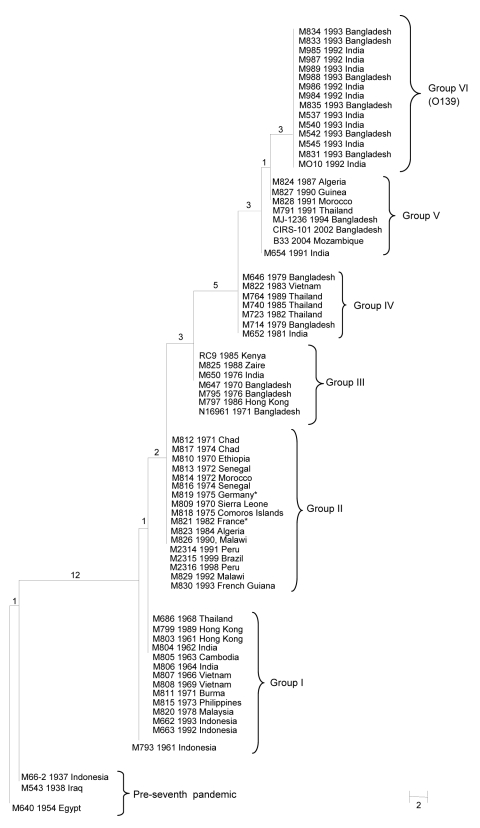
Maximum-parsimony tree of 68 seventh cholera pandemic and 3 pre–seventh cholera pandemic isolates. The tree was based on 18 N16961 seventh pandemic single-nucleotide polymorphisms (SNPs) and 12 MO10 O139 SNPs. The 3 pre–seventh pandemic isolates were used as an outgroup. Each strain name is followed by the year and location of isolation. All 15 O139 isolates had the same SNP profile and are shown as group VI. The numbers on each node represent the number of supporting SNPs. M821 and M819 from France and Germany are likely imported from either Africa or Asia. SNP data for the following isolates were obtained from GenBank: accession nos. RC9, ACHX00000000; MJ-1236, CP001385/CP001486; B33, ACHZ00000000; CIRS 101, ACVA00000000; MO10, AAKF00000000; N16961, AE003852; and M66–2, CP001233. Scale bar indicates number of nucleotide substitutions.

## Conclusions

The presence of isolates from Africa in 3 groups can be explained by multiple introductions of cholera into Africa from cholera-endemic regions in Asia. The isolates in the first introduction in the 1970s shared a single origin (group II). However, during the late 1980s and early 1990s, cholera outbreaks appeared to be caused by strains from 3 related sources. The first source came from group II, which was already established in Africa, and the second and third sources came from groups III and V in Asia. Because both groups were supported by multiple SNPs, it is less likely that the 1970s isolates from Africa and Asia evolved in parallel to fall into the same groups. Additionally, B33 in group V carries a classical CTX prophage (*8*), which indicates that this strain likely originated in Asia.

The cholera epidemic in Latin America was originally suspected to have come from Asia and to have been facilitated by the discharge of contaminated ballast water into Peruvian ports by international trade ships (*2*). However, the isolates from Latin America analyzed in this study were closely related to isolates found in Africa in the 1970s and 1990s. Four isolates, 2 from Peru and 1 each from Brazil and French Guiana, had an SNP profile identical to the 12 isolates from Africa that originated during that period. No isolates from Asia fell into this group. This finding suggests that the strain that caused the epidemic in Latin America came from Africa rather than Asia.

The outbreak in Peru occurred in parallel with the upsurge of cholera generally in Africa (*1*) and could have been imported at that time. However, the epidemic strain may have reached Latin America well before it caused the epidemic in 1990s, given the ability of the organism to persist in the marine environment for long periods (*2*). The strain could have been brought into the region during the mass migration from Africa to Latin America in the 1970s (*9*). The isolates from Latin America differ by 1 locus from the other seventh pandemic strains (Asia and Africa) by multilocus enzyme electrophoresis (*10*) and also differ in the *Vibrio* spp. seventh pandemic island-II gene cluster (*11*), which suggests that further evolution occurred after the strain separated from its likely ancestral strain from Africa and supports this latter scenario. The epidemic strain in Latin America could not have originated from the 1990s isolates from Asia in Groups III–V because they arose later than Group II isolates. However, a 1970s lineage in Asia that spread to Africa may have remained in Asia until the 1990s but was not represented in the isolates sampled. Further investigation is needed to resolve this hypothesis. Furthermore, although the SNP profiles of the isolates from Africa and Latin America are identical, they may have diverged substantially because the SNPs used can only determine node positions but not branch length caused by phylogenetic discovery bias (*12*).

Our SNP data clearly show that O139 Bengal was a derivative of the seventh pandemic, as previously suggested (*13*). Nine of the 12 O139 SNPs can now be seen to have arisen in its O1 precursor strain because they were present in seventh pandemic isolates as early as 1979 (Technical Appendix). These SNPs also resolved the relationships of Groups IV–V. Some studies have suggested that the O139 variant may have multiple origins (*14*). However, our results suggest that these O139 isolates from the then new epidemic have a single origin, which is consistent with earlier ribotyping data (*15*).

Our data show each of the groups/genotypes spread easily and widely to multiple countries or regions. This finding suggests that cholera epidemics or upsurges, which often occurred at the same time in many countries, were caused by the spread of newly arisen genotypes. Additionally, a genotype can also persist for long periods. Thus, in cholera-endemic regions such as Southeast Asia and Africa, cholera can be caused not only by an endemic genotype, but also by new epidemic genotypes. This finding is useful for control of cholera epidemics.

## Supplementary Material

Technical Appendix Methods: Primers and Location of Single Nucleotide Polymorphisms studied, Hairpin Real-Time PCR (HP RT-PCR).

## Appendix

**Table A1:** Single nucleotide polymorphism profiles of 71 isolates of pandemic *Vibrio cholerae**

Group	SNP profile (no. isolates)	N16961 SNPs†*																			MO10 SNPs†											
		vc 0672	vc 0835	vc 0837	vc 0987	vc 1088	vc 1091	vc 1248	vca 0946	vc 2046	vc 2080	vc 2091	vc 2674	vc 1967	vca 0247	vca 1073	vc 0329	vc 1579	vc 1898		vc 2363	vc 2562	vc 1877	vc 0959	vc 1082	vc 1865	vc 2077	vc 1318	vc 0008	vc 0847	vc 1707	vc 2599
Pre-7th	1 (2)	T	A	G	C	A	C	C	T	G	C	G	G	A	C	C	C	C	G		A	C	C	C	C	T	C	A	G	G	G	C
	2 (1)	T	A	G	C	A	C	C	T	G	C	G	G	A	C	C	C	C	A		A	C	C	C	C	T	C	A	G	G	G	C
I	3 (1)	G	G	A	T	T	T	T	C	A	T	T	A	A	C	C	C	C	G		A	C	C	C	C	T	C	A	G	G	G	C
	4 (13)	G	G	A	T	T	T	T	C	A	T	T	A	T	C	C	C	C	G		A	C	C	C	C	T	C	A	G	G	G	C
II	5 (17)	G	G	A	T	T	T	T	C	A	T	T	A	T	T	T	C	C	G		A	C	C	C	C	T	C	A	G	G	G	C
III	6 (7)	G	G	A	T	T	T	T	C	A	T	T	A	T	T	T	T	T	A		A	C	C	C	C	T	C	A	G	G	G	C
IV	7 (7)	G	G	A	T	T	T	T	C	A	T	T	A	T	T	T	T	T	A		G	T	A	T	T	T	C	A	G	G	G	C
V	8 (1)	G	G	A	T	T	T	T	C	A	T	T	A	T	T	T	T	T	A		G	T	A	T	T	G	T	T	G	G	G	C
	9 (7)	G	G	A	T	T	T	T	C	A	T	T	A	T	T	T	T	T	A		G	T	A	T	T	G	T	T	A	G	G	C
VI	10 (15)	G	G	A	T	T	T	T	C	A	T	T	A	T	T	T	T	T	A		G	T	A	T	T	G	T	T	A	A	A	T

*SNP, single-nucleotide polymorphism. Complete details for each strain and SNP profile can be found in the Technical Appendix. SNP data for 7 isolates were obtained from GenBank (accession nos. RC9- ACHX00000000, MJ-1236- CP001385/CP001486, B33-ACHZ00000000, CIRS 101-ACVA00000000, MO10- AAKF00000000, N16961-AE003852, M66-2-CP001233). †SNPs were selected from a comparison of N16961 with M66-2, and N16961 with MO10. The exact location of each SNP can be found in the Technical Appendix. SNP mutations are shaded; ancestral SNPs have been left unshaded. The SNPs are grouped in the order in which the mutations are inferred to have occurred.
