# Supplementary material for: Evolution of Seventh Cholera Pandemic and Origin of 1991 Epidemic, Latin America
Source: Emerg Infect Dis. 2010 Jul;16(7):1130–2. doi: 10.3201/eid1607.100131 (PMC3321917; doi:10.3201/eid1607.100131)
Supplement: Technical Appendix — Methods: Primers and Location of Single Nucleotide Polymorphisms studied, Hairpin Real-Time PCR (HP RT-PCR). [file 10-0131-Techapp.pdf]

DOI: 10.3201/eid1607.100131

Evolution of Seventh Cholera Pandemic and Origin of 1991 Epidemic, Latin America

Technical Appendix

Methods

Primers and Location of Single Nucleotide Polymorphisms studied

The detection of each single nucleotide polymorphism (SNP) required 2 forward primers. The first forward primer contained the SNP of the seventh pandemic at the 3′end, while the second primer contained the SNP of either MO10 O139 Bengal or M66-2 pre–seventh pandemic. A complementary tail was added to the 5′end of each forward primer to form a hairpin structure. Alterations to the original primer sequence were also made to facilitate the folding of the primer into a hairpin structure. Table A-1 and Table A-2 contain the name, location, forward and reverse primer for each SNP. The same reverse primer was used for each pair of SNP reactions.

Hairpin Real-Time PCR (HP RT-PCR)

All RT-PCRs were carried out in a Rotor-Gene 6000 instrument (Corbett Life Science, Mortlake, New South Wales, Australia) with a 72-well rotor disk. Each RT-PCR reaction consisted of ≈100 ng DNA, 2.5 μmol each of forward and reverse primers and 5 μL SensiMix*Plus* SYBR Green (Quantace, Alexandria, New South Wales, Australia) (includes 2× Mix containing reaction buffer), Heat- Activated Taq DNA polymerase, dNTPs 6 mM MgCl2, SYBR Green I. MilliQ water was added to adjust the final volume to 10 μL. The thermal cycling conditions were set up as follows: stage 1, 95ºC for 10 min to activate Taq polymerase, stage 2, 95ºC for 15 s, 69ºC for 30 s, repeated 10 × followed by stage 3, 95ºC for 15 s, 60ºC for 30 s, repeated 40 ×. On completion of each run, data were collected and analyzed with Rotor-Gene operating software v1.7.87 (Corbett Life Science). The fluorescent signal for each reaction was measured at the end of each cycle and plotted on a fluorescence curve. The cycle threshold (Ct) was set across the amplification curves during the exponential fluorescence phase.

Table A-1: Name, location and primers of 7th cholera pandemic and pre–7th pandemic single-nucleotide polymorphisms*

Locus	Gene	Annotation	Location in N16961	7th Pandemic		M66-2		Reverse primer (5'→ 3')
				Forward primer (5'→ 3')	SNP	Forward primer (5'→3')	SNP	
Large chromosome								
vc0329	*rpoC*	DNA-directed RNA polymerase, beta subunit	393808	atctttacc***T***GTCCTGCAGCAGGTAAAGA**T**	T	gtctttacc***T***GTCCTGCAGCAGGTAAAGA**C**	C	GAAGTATTGAGCTGGCATGTC
vc0672	*ptsP*	Phosphoenolpyruvate-protein phosphotransferase	765286	ttctctac***C***GCCTTGGCAGTAGAGA**A**	A	ctctctac***C***GCCTTGGCAGTAGAGA**G**	G	TCCCCATCGAGCTTTTTA
vc0835	*tcpT*	Toxin co-regulated pilus biosynthesis protein T	943582	caaacattAGCCGA***C***GCGCTAATGTTT**G**	G	aaaacattAGCCGA***C***GCGCTAATGTTT**T**	T	TGAGGTAGTTTTCGGTTCCACG
vc0837	*tcpF*	Toxin co-regulated pilus biosynthesis protein F	946533	ccactag***G***AACCATATCAGCCTAGTG**G**	G	tcactag***G***AACCATATCAGCCTAGTG**A**	A	GTATTTGACCACTTGTAACCAT
vc0987	*hemH*	Ferrochelatase	1099038	ttgcagc***G***AGGAAGAGTGGCTGCA**A**	A	ctgcagc***G***AGGAAGAGTGGCTGCA**G**	G	TTTTAATGCCTTGGCGAG
vc1088		Sensor histidine kinase	1202761	agacaaA***G***CACTCGGGTGCCTTGTC**T**	T	ggacaaA***G***CACTCGGGTGCCTTGTC**C**	C	GCCTACAGCAGCATCAAAAAAT
vc1091	*oppA*	Oligopeptide ABC transporter, periplasmic oligopeptide-binding protein	1206798	agcggtA***T***TCCGGAAATCACCGC**T**	T	tgcggtA***T***TCCGGAAATCACCGC**A**	A	CTTCGCTTCTGCAATACGCTCT
vc1248		Methyl-accepting chemotaxis protein	1435093	aacgctc***A***ACCTATCATAACGAGCGT**T**	T	gacgctc***A***ACCTATCATAACGAGCGT**C**	C	GGCTCAGGCATGTTGTTGG
vc1579		Enterobactin synthetase component F-related protein	1770713	atcaccatcT***G***CCGTTAATTGATGGTGA**T**	T	gtcaccatcT***G***CCGTTAATTGATGGTGA**C**	C	TGCTTGTTTATATGTTGTGCCT
vc1898		Methyl-accepting chemotaxis protein	2128112	aatgatC***A***GTTTTGCGCTGATCAT**T**	T	gatgatC***A***GTTTTGCGCTGATCAT**C**	C	AATGCAGCGGTTGAAACACT
vc1967		Methyl-accepting chemotaxis protein	2201875	tgtttcttG***C***ATCAACAATCAAGAAAC**A**	A	cgtttcttG***C***ATCAACAATCAAGAAAC**G**	G	ACTTGCGTTGCTTTATCGTAGG
vc2046		Conserved hypothetical protein	2287749	aagcttt***G***TGGCCAGTGCAAAGCT**T**	T	gagcttt***G***TGGCCAGTGCAAAGCT**C**	C	AACCTTGAGTATCCTGTGG
vc2080		Transcriptional regulator, AraC/XylS family	2322891	agctctt***A***TCTCCATCCGAGTTAAGAGC**T**	T	cgctctt***A***TCTCCATCCGAGTTAAGAGC**G**	G	GCATTATCTAACGACGGA
vc2091	*sdhC*	Succinate dehydrogenase, cytochrome b556 subunit	2336169	actctcC***T***GACAGGTGAGGAGAG**T**	T	tctctcC***T***GACAGGTGAGGAGAG**A**	A	CAGCAATCGCATCCATCCT
vc2674	*hslU*	Protease HslVU, ATPase subunit HslU	2927259	tgcctt***T***AACCTTGATGAAAGGC**A**	A	cgcctt***T***AACCTTGATGAAAGGC**G**	G	TGTTGAAGTGACCCCGAAA
**Small chromosome**								
vca0247		Transcriptional regulator, DeoR family	302905	actttgc***G***ATACAATCGAGCGCAAAG**T**	T	gctttgc***G***ATACAATCGAGCGCAAAG**C**	C	TCGGTTAGCCCTTTGCCAGA
vca0946	*malK*	Maltose/maltodextrin ABC transporter, ATP-binding protein	830773	gccgctaGAACCGTCCAA***T***AGCGG**C**	C	accgctaGAACCGTCCAA***T***AGCGG**T**	T	GTCGTACAAATTGAGGTGCGGG
vca1073		Bifunctional protein putA	956775	agtgtg***C***GATGCAGATGTGGCACAC**T**	T	ggtgtg***C***GATGCAGATGTGGCACAC**C**	C	ACTGCGTGTGCTGTTTGT

*SNP, single nucleotide polymorphism. Nucleotides in lower case indicate a complementary tail which was added to the primer to form a hairpin structure.
Bold and italic nucleotides indicate that a deliberate change has been made to the sequence to facilitate the folding of the primer into a hairpin structure.
Bold and underlined nucleotides indicate the corresponding SNP of either the 7th pandemic or M-662.

Table A-2: Name, location and primers of the *Vibrio cholerae* O139 MO10 single-nucleotide polymorphisms*

Locus	Gene	Annotation	Location in N16961	7th pandemic Forward primer (5'→3')	7th pandemic SNP	MO10 Forward primer (5'→3')	MO10 SNP	Reverse primer (5'→3')
vc0008		Amino acid ABC transporter ATP binding protein	5414	cgatacc***C***GGTATGTTTTTGGGTATC**G**	G	tgatacc***C***GGTATGTTTTTGGGTATC**A**	A	GCGTGAACTTTCTTGAGC
vc0847		Phage family integrase	913179	caacagc***C***TTGCCGTTTGGCTGTT**G**	G	taacagc***C***TTGCCGTTTGGCTGTT**A**	A	GCCATCGTGATTTTATTT
vc0959		Haemolysin (putative)	1024406	gccgaaAG***T***TCTTGGCGATCTTTCGG**C**	C	accgaaAG***T***TCTTGGCGATCTTTCGG**T**	T	GGTCCGAGTAGAAAGTCC
vc1082		Hypothetical protein	1149897	ggcctt***C***TTCTGGTTGAGAAGGC**C**	C	agcctt***C***TTCTGGTTGAGAAGGC**T**	T	AGATGGGCAAATACCTTA
vc1318	*ompV*	outer membrane protein OmpV	1401874	tggcaaC***A***ATATCGCCTGTGTTGCC**A**	A	aggcaaC***A***ATATCGCCTGTGTTGCC**T**	T	TACCAGCAAGGGCACAATCA
vc1707		Hypothetical protein	1838824	cgtttgga***A***CTGTCACATTCCAAAC**G**	G	tgtttgga***A***CTGTCACATTCCAAAC**A**	A	AAACTTCGATAGCGTGAT
vc1865		Hypothetical protein	2005889	accagcA***A***TTTAACTTGCGCTGG**T**	T	cccagcA***A***TTTAACTTGCGCTGG**G**	G	CCCGCACCCAAGGCAAGC
vc1877	*1pxk*	Tetra-acyldisaccharide 4'-kinase	2021732	gtcgtg***G***ATGTTACCCACCACGA**C**	C	ttcgtg***G***ATGTTACCCACCACGA**A**	A	TATCAAACGGGCGACAAA
vc2077		Ferrous iron transport protein B	2234253	gcacacC***C***TCTGCATCAGGTGTG**C**	C	acacacC***C***TCTGCATCAGGTGTG**T**	T	AAAGAAGCGGTTGTGGGG
vc2362		Threonine Synthetase	2518900	ttttttcC***G***ATTGTGCCGGAAAAA**A**	A	ctttttcC***G***ATTGTGCCGGAAAAA**G**	G	GGTCAAGCCGTTCGCCAA
vc2562	*cpdB*	Bi-functional 2' 3'- cyclic nucleotide 2'-phosphodiesterase/3' nucleotidase periplasmic precursor protein	2744542	gtgtacctt***G***CGATCATCAAGGTACA**C**	C	atgtacctt***G***CGATCATCAAGGTACA**T**	T	ACATCACGTCGTTCGCTT
vc2599		Ribonuclease R	2766113	gtgaagG***C***TTGCTACGGCCTTCA**C**	C	atgaagG***C***TTGCTACGGCCTTCA**T**	T	CACCAACGAAATCAGAGT

*SNP, single nucleotide polymorphism. Nucleotides in lower case indicate a complementary tail which was added to the primer to form a hairpin structure.
Bold and italic nucleotides indicate that a deliberate change has been made to the sequence to facilitate the folding of the primer into a hairpin structure. Bold and underlined nucleotides indicate the corresponding SNP of either the 7th pandemic or MO10.

Table A-3: Single-nucleotide polymorphism (SNP) profiles of 71 isolates of pandemic *Vibrio cholerae*

					N16961 SNPs																		MO10 SNPs											
Group	SNP profile	Isolate	Year	Location	vc0672	vc0835	vc0837	vc0987	vc1088	vc1091	vc1248	vca0946	vc2046	vc2080	vc2091	vc2674	vc1967	vca0247	vca1073	vc0329	vc1579	vc1898	vc2363	vc2562	vc1877	vc0959	vc1082	vc1865	vc2077	vc1318	vc0008	vc0847	vc1707	vc2599
**Pre-7th**	**1**	M66-2†	1937	Indonesia	T	A	G	C	A	C	C	T	G	C	G	G	A	C	C	C	C	G	A	C	C	C	C	T	C	A	G	G	G	C
		M543	1938	Iraq	T	A	G	C	A	C	C	T	G	C	G	G	A	C	C	C	C	G	A	C	C	C	C	T	C	A	G	G	G	C
	**2**	M640	1954	Egypt	T	A	G	C	A	C	C	T	G	C	G	G	A	C	C	C	C	A	A	C	C	C	C	T	C	A	G	G	G	C
**I**	**3**	M793	1961	Indonesia	G	G	A	T	T	T	T	C	A	T	T	A	A	C	C	C	C	G	A	C	C	C	C	T	C	A	G	G	G	C
	**4**	M686	1968	Thailand	G	G	A	T	T	T	T	C	A	T	T	A	T	C	C	C	C	G	A	C	C	C	C	T	C	A	G	G	G	C
		M799	1989	Hong Kong	G	G	A	T	T	T	T	C	A	T	T	A	T	C	C	C	C	G	A	C	C	C	C	T	C	A	G	G	G	C
		M803	1961	Hong Kong	G	G	A	T	T	T	T	C	A	T	T	A	T	C	C	C	C	G	A	C	C	C	C	T	C	A	G	G	G	C
		M804	1962	India	G	G	A	T	T	T	T	C	A	T	T	A	T	C	C	C	C	G	A	C	C	C	C	T	C	A	G	G	G	C
		M805	1963	Cambodia	G	G	A	T	T	T	T	C	A	T	T	A	T	C	C	C	C	G	A	C	C	C	C	T	C	A	G	G	G	C
		M806	1964	India	G	G	A	T	T	T	T	C	A	T	T	A	T	C	C	C	C	G	A	C	C	C	C	T	C	A	G	G	G	C
		M807	1966	Vietnam	G	G	A	T	T	T	T	C	A	T	T	A	T	C	C	C	C	G	A	C	C	C	C	T	C	A	G	G	G	C
		M808	1969	Vietnam	G	G	A	T	T	T	T	C	A	T	T	A	T	C	C	C	C	G	A	C	C	C	C	T	C	A	G	G	G	C

		M811	1971	Burma	G	G	A	T	T	T	T	C	A	T	T	A	T	C	C	C	C	G	A	C	C	C	C	T	C	A	G	G	G	C
		M815	1973	Philippines	G	G	A	T	T	T	T	C	A	T	T	A	T	C	C	C	C	G	A	C	C	C	C	T	C	A	G	G	G	C
		M820	1978	Malaysia	G	G	A	T	T	T	T	C	A	T	T	A	T	C	C	C	C	G	A	C	C	C	C	T	C	A	G	G	G	C
		M662	1993	Indonesia	G	G	A	T	T	T	T	C	A	T	T	A	T	C	C	C	C	G	A	C	C	C	C	T	C	A	G	G	G	C
		M663	1992	Indonesia	G	G	A	T	T	T	T	C	A	T	T	A	T	C	C	C	C	G	A	C	C	C	C	T	C	A	G	G	G	C
**II**	**5**	M809	1970	Sierra Leone	G	G	A	T	T	T	T	C	A	T	T	A	T	T	T	C	C	G	A	C	C	C	C	T	C	A	G	G	G	C
		M821	1982	France	G	G	A	T	T	T	T	C	A	T	T	A	T	T	T	C	C	G	A	C	C	C	C	T	C	A	G	G	G	C
		M823	1984	Algeria	G	G	A	T	T	T	T	C	A	T	T	A	T	T	T	C	C	G	A	C	C	C	C	T	C	A	G	G	G	C
		M826	1990	Malawi	G	G	A	T	T	T	T	C	A	T	T	A	T	T	T	C	C	G	A	C	C	C	C	T	C	A	G	G	G	C
		M2314	1991	Peru	G	G	A	T	T	T	T	C	A	T	T	A	T	T	T	C	C	G	A	C	C	C	C	T	C	A	G	G	G	C
		M2315	1999	Brazil	G	G	A	T	T	T	T	C	A	T	T	A	T	T	T	C	C	G	A	C	C	C	C	T	C	A	G	G	G	C
		M2316	1998	Peru	G	G	A	T	T	T	T	C	A	T	T	A	T	T	T	C	C	G	A	C	C	C	C	T	C	A	G	G	G	C
		M829	1992	Malawi	G	G	A	T	T	T	T	C	A	T	T	A	T	T	T	C	C	G	A	C	C	C	C	T	C	A	G	G	G	C
		M830	1993	French Guiana	G	G	A	T	T	T	T	C	A	T	T	A	T	T	T	C	C	G	A	C	C	C	C	T	C	A	G	G	G	C
		M812	1971	Chad	G	G	A	T	T	T	T	C	A	T	T	A	T	T	T	C	C	G	A	C	C	C	C	T	C	A	G	G	G	C
		M817	1974	Chad	G	G	A	T	T	T	T	C	A	T	T	A	T	T	T	C	C	G	A	C	C	C	C	T	C	A	G	G	G	C
		M810	1970	Ethiopia	G	G	A	T	T	T	T	C	A	T	T	A	T	T	T	C	C	G	A	C	C	C	C	T	C	A	G	G	G	C
		M813	1972	Senegal	G	G	A	T	T	T	T	C	A	T	T	A	T	T	T	C	C	G	A	C	C	C	C	T	C	A	G	G	G	C
		M814	1972	Morocco	G	G	A	T	T	T	T	C	A	T	T	A	T	T	T	C	C	G	A	C	C	C	C	T	C	A	G	G	G	C
		M816	1974	Senegal	G	G	A	T	T	T	T	C	A	T	T	A	T	T	T	C	C	G	A	C	C	C	C	T	C	A	G	G	G	C
		M819	1975	Germany	G	G	A	T	T	T	T	C	A	T	T	A	T	T	T	C	C	G	A	C	C	C	C	T	C	A	G	G	G	C
		M818	1975	Comoros Islands	G	G	A	T	T	T	T	C	A	T	T	A	T	T	T	C	C	G	A	C	C	C	C	T	C	A	G	G	G	C
**III**	**6**	M650	1976	India	G	G	A	T	T	T	T	C	A	T	T	A	T	T	T	T	T	A	A	C	C	C	C	T	C	A	G	G	G	C
		M647	1970	Bangladesh	G	G	A	T	T	T	T	C	A	T	T	A	T	T	T	T	T	A	A	C	C	C	C	T	C	A	G	G	G	C
		M795	1976	Bangladesh	G	G	A	T	T	T	T	C	A	T	T	A	T	T	T	T	T	A	A	C	C	C	C	T	C	A	G	G	G	C
		M797	1986	Hong Kong	G	G	A	T	T	T	T	C	A	T	T	A	T	T	T	T	T	A	A	C	C	C	C	T	C	A	G	G	G	C
		N16961†	1971	Bangladesh	G	G	A	T	T	T	T	C	A	T	T	A	T	T	T	T	T	A	A	C	C	C	C	T	C	A	G	G	G	C
		RC9†	1985	Kenya	G	G	A	T	T	T	T	C	A	T	T	A	T	T	T	T	T	A	A	C	C	C	C	T	C	A	G	G	G	C
		M825	1988	Zaire	G	G	A	T	T	T	T	C	A	T	T	A	T	T	T	T	T	A	A	C	C	C	C	T	C	A	G	G	G	C
**IV**	**7**	M646	1979	Bangladesh	G	G	A	T	T	T	T	C	A	T	T	A	T	T	T	T	T	A	G	T	A	T	T	T	C	A	G	G	G	C
		M652	1981	India	G	G	A	T	T	T	T	C	A	T	T	A	T	T	T	T	T	A	G	T	A	T	T	T	C	A	G	G	G	C
		M714	1979	Bangladesh	G	G	A	T	T	T	T	C	A	T	T	A	T	T	T	T	T	A	G	T	A	T	T	T	C	A	G	G	G	C
		M723	1982	Thailand	G	G	A	T	T	T	T	C	A	T	T	A	T	T	T	T	T	A	G	T	A	T	T	T	C	A	G	G	G	C
		M740	1985	Thailand	G	G	A	T	T	T	T	C	A	T	T	A	T	T	T	T	T	A	G	T	A	T	T	T	C	A	G	G	G	C
		M764	1989	Thailand	G	G	A	T	T	T	T	C	A	T	T	A	T	T	T	T	T	A	G	T	A	T	T	T	C	A	G	G	G	C
		M822	1983	Vietnam	G	G	A	T	T	T	T	C	A	T	T	A	T	T	T	T	T	A	G	T	A	T	T	T	C	A	G	G	G	C
**V**	**8**	M654	1991	India	G	G	A	T	T	T	T	C	A	T	T	A	T	T	T	T	T	A	G	T	A	T	T	G	T	T	G	G	G	C
	**9**	M791	1991	Thailand	G	G	A	T	T	T	T	C	A	T	T	A	T	T	T	T	T	A	G	T	A	T	T	G	T	T	A	G	G	C

		M824	1987	Algeria	G	G	A	T	T	T	T	C	A	T	T	A	T	T	T	T	T	A	G	T	A	T	T	G	T	T	A	G	G	C
		MJ1236†	1994	Bangladesh	G	G	A	T	T	T	T	C	A	T	T	A	T	T	T	T	T	A	G	T	A	T	T	G	T	T	A	G	G	C
		CIRS101 †	2002	Bangladesh	G	G	A	T	T	T	T	C	A	T	T	A	T	T	T	T	T	A	G	T	A	T	T	G	T	T	A	G	G	C
		B33†	2004	Mozambique	G	G	A	T	T	T	T	C	A	T	T	A	T	T	T	T	T	A	G	T	A	T	T	G	T	T	A	G	G	C
		M827	1990	Guinea	G	G	A	T	T	T	T	C	A	T	T	A	T	T	T	T	T	A	G	T	A	T	T	G	T	T	A	G	G	C
		M828	1991	Morrocco	G	G	A	T	T	T	T	C	A	T	T	A	T	T	T	T	T	A	G	T	A	T	T	G	T	T	A	G	G	C
**VI**	**10**	M834	1993	Bangladesh	G	G	A	T	T	T	T	C	A	T	T	A	T	T	T	T	T	A	G	T	A	T	T	G	T	T	A	A	A	T
		M833	1993	Bangladesh	G	G	A	T	T	T	T	C	A	T	T	A	T	T	T	T	T	A	G	T	A	T	T	G	T	T	A	A	A	T
		M985	1992	India	G	G	A	T	T	T	T	C	A	T	T	A	T	T	T	T	T	A	G	T	A	T	T	G	T	T	A	A	A	T
		M987	1992	India	G	G	A	T	T	T	T	C	A	T	T	A	T	T	T	T	T	A	G	T	A	T	T	G	T	T	A	A	A	T
		M989	1993	India	G	G	A	T	T	T	T	C	A	T	T	A	T	T	T	T	T	A	G	T	A	T	T	G	T	T	A	A	A	T
		M988	1993	Bangladesh	G	G	A	T	T	T	T	C	A	T	T	A	T	T	T	T	T	A	G	T	A	T	T	G	T	T	A	A	A	T
		M986	1992	India	G	G	A	T	T	T	T	C	A	T	T	A	T	T	T	T	T	A	G	T	A	T	T	G	T	T	A	A	A	T
		M984	1992	India	G	G	A	T	T	T	T	C	A	T	T	A	T	T	T	T	T	A	G	T	A	T	T	G	T	T	A	A	A	T
		M835	1993	Bangladesh	G	G	A	T	T	T	T	C	A	T	T	A	T	T	T	T	T	A	G	T	A	T	T	G	T	T	A	A	A	T
		M537	1993	India	G	G	A	T	T	T	T	C	A	T	T	A	T	T	T	T	T	A	G	T	A	T	T	G	T	T	A	A	A	T
		M540	1993	India	G	G	A	T	T	T	T	C	A	T	T	A	T	T	T	T	T	A	G	T	A	T	T	G	T	T	A	A	A	T
		M542	1993	Bangladesh	G	G	A	T	T	T	T	C	A	T	T	A	T	T	T	T	T	A	G	T	A	T	T	G	T	T	A	A	A	T
		M545	1993	India	G	G	A	T	T	T	T	C	A	T	T	A	T	T	T	T	T	A	G	T	A	T	T	G	T	T	A	A	A	T
		M831	1993	Bangladesh	G	G	A	T	T	T	T	C	A	T	T	A	T	T	T	T	T	A	G	T	A	T	T	G	T	T	A	A	A	T
		MO10†	1992	India	G	G	A	T	T	T	T	C	A	T	T	A	T	T	T	T	T	A	G	T	A	T	T	G	T	T	A	A	A	T

§ SNPs were selected from comparison between N16961 with M66-2 ,and N16961 with MO10. SNP mutations are shaded in blue, ancestral SNPs have been left unshaded. The SNPs are grouped in the order in which the mutations are inferred to have occurred. Horizontal lines separate indivdual SNP profiles

† SNP data for these isolates was obtained from GenBank.(accession nos: RC9- ACHX00000000, MJ-1236- CP001385/CP001486, B33- ACHZ00000000, CIRS 101- ACVA00000000, MO10- AAKF00000000, N16961- AE003852, M66-2- CP001233)
